# Supplementary material for: Vitamin D Modified DSS-Induced Colitis in Mice via STING Signaling Pathway
Source: Biology (Basel). 2025 Jun 18;14(6):715. doi: 10.3390/biology14060715 (PMC12190092; doi:10.3390/biology14060715)
Supplement: Supplementary file 1 [file biology-14-00715-s001.zip › Supplementary Materials S1: 16s-report of gut microbiota/Groups/diff_analysis/Correlation/Genus-EC.pdf]

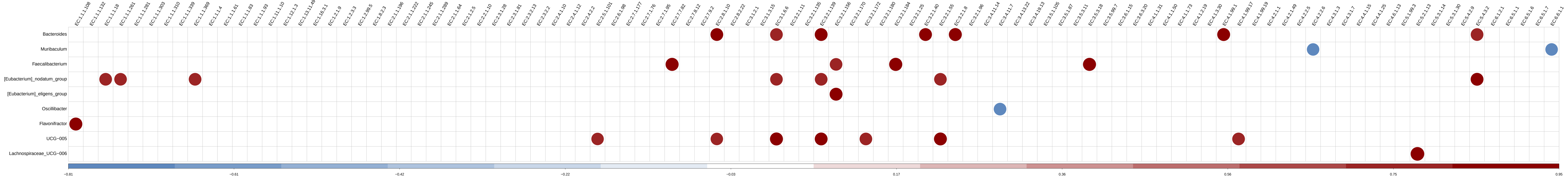
EC:1.1.1.108
EC:1.1.1.132
EC:1.1.1.18
EC:1.1.1.261
EC:1.1.1.281
EC:1.1.1.303
EC:1.1.1.310
EC:1.1.1.339
EC:1.1.1.369
EC:1.1.1.4
EC:1.1.1.61
EC:1.1.1.83
EC:1.1.1.93
EC:1.11.1.10
EC:1.12.1.3
EC:1.13.11.49
EC:1.16.3.1
EC:1.2.1.9
EC:1.2.3.3
EC:1.2.99.5
EC:1.8.2.3
EC:2.1.1.196
EC:2.1.1.222
EC:2.1.1.245
EC:2.1.1.289
EC:2.1.1.64
EC:2.1.2.5
EC:2.2.1.10
EC:2.3.1.28
EC:2.3.1.81
EC:2.3.2.13
EC:2.3.2.2
EC:2.4.1.10
EC:2.4.1.12
EC:2.4.2.2
EC:2.5.1.101
EC:2.6.1.98
EC:2.7.1.177
EC:2.7.1.76
EC:2.7.1.95
EC:2.7.7.82
EC:2.7.8.12
EC:2.7.9.2
EC:2.8.1.10
EC:2.8.2.22
EC:3.1.2.1
EC:3.1.3.15
EC:3.1.6.6
EC:3.2.1.11
EC:3.2.1.135
EC:3.2.1.139
EC:3.2.1.156
EC:3.2.1.170
EC:3.2.1.172
EC:3.2.1.180
EC:3.2.1.184
EC:3.2.1.25
EC:3.2.1.40
EC:3.2.1.55
EC:3.2.1.8
EC:3.2.1.96
EC:3.4.11.14
EC:3.4.11.7
EC:3.4.13.22
EC:3.4.19.13
EC:3.5.1.105
EC:3.5.1.87
EC:3.5.3.11
EC:3.5.3.18
EC:3.5.99.7
EC:3.6.1.15
EC:3.6.3.20
EC:4.1.1.31
EC:4.1.1.50
EC:4.1.1.73
EC:4.1.2.19
EC:4.1.3.30
EC:4.1.99.1
EC:4.1.99.17
EC:4.1.99.19
EC:4.2.1.1
EC:4.2.1.49
EC:4.2.2.5
EC:4.2.2.6
EC:4.3.1.3
EC:4.3.1.7
EC:4.4.1.15
EC:4.4.1.25
EC:4.6.1.13
EC:5.1.99.3
EC:5.2.1.13
EC:5.3.1.14
EC:5.3.1.30
EC:5.4.2.9
EC:5.4.3.2
EC:6.1.2.1
EC:6.5.1.1
EC:6.5.1.6
EC:6.5.1.7
EC:6.6.1.1
Bacteroides
Muribaculum
Faecalibacterium
[Eubacterium]_nodatum_group
[Eubacterium]_eligens_group
Oscillibacter
Flavonifractor
UCG−005
Lachnospiraceae_UCG−006
−0.81
−0.61
−0.42
−0.22
−0.03
0.17
0.36
0.56
0.75
0.95
